# A Highly Unusual Source and Course of the Inferior Gluteal Nerve With Related Variations: A Case Report

**DOI:** 10.7759/cureus.48694

**Published:** 2023-11-12

**Authors:** Emma R Lesser, Mitchell Kilgore, Joe Iwanaga, Anna Carrera, Francisco Reina, Aaron S Dumont, R. Shane Tubbs

**Affiliations:** 1 Department of Neurosurgery, Tulane University School of Medicine, New Orleans, USA; 2 Department of Medical Sciences; Clinical Anatomy, Embryology and Neurosciences Research Group (NEOMA), University of Girona, Girona, ESP; 3 Anatomical Sciences, St. George's University, St. George's, GRD; 4 Neurosurgery and Ochsner Neuroscience Institute, Ochsner Medical System, New Orleans, USA

**Keywords:** variations, sacral plexus, sciatic nerve, cadaver, anatomy

## Abstract

The gluteal region is rich in neurovasculature that divides in predictable ways. Though these characteristic relationships can be helpful when orienting oneself to the gluteal region, it is especially useful to be aware of anatomical variations in this region. Knowledge of such differences allows for a better appreciation of nerve entrapments and neuropathies, and such awareness is critical during surgeries. In this case report, we explore an uncharacteristic relationship among the piriformis muscle, the sciatic nerve and its components, and the inferior gluteal and posterior femoral cutaneous nerves.

## Introduction

The sciatic nerve (SN) and inferior gluteal nerve (IGN) are components of the sacral plexus and provide significant sensorimotor innervations to the lower limbs. The SN arises from the anterior rami of L4-S3 and typically exits the pelvis inferior to the piriformis muscle (PM) via the greater sciatic foramen prior to bifurcating into the tibial nerve (TN) and common fibular nerve (CFN) in the thigh [1]. The IGN receives contributions from L5-S2; it branches from the proximal SN and accompanies it through the greater sciatic foramen before dividing into terminal branches that provide motor innervation to the gluteus maximus muscle (GMax) and relay sensation from the skin of the hip [2]. The dense musculoskeletal anatomy of the pelvic region leaves these nerves susceptible to compression, mechanical entrapment, and iatrogenic injury during lumbopelvic injections and surgical procedures. The resulting entrapment can result in sensorimotor deficits in the regions of the lower limbs they innervate, with consequences for patient quality of life [1-3].

The IGN normally leaves the pelvic cavity through the greater sciatic foramen along with the SN and passes deeply to the PM, usually along with the inferior gluteal vasculature. After emerging inferiorly to the PM, the IGN reverses its course folds on itself and travels superficially to provide motor innervation to the GMax. Genetic and developmental factors and developmental anomalies, such as neural tube defects, can disrupt normal muscular development in this region [4]. For this reason, it is important to appreciate variations.

Variants of the proximal IGN, particularly in relation to the PM, have not been thoroughly described. In the extant literature, few studies have reported variants ranging from total absence of the nerve [5] to it following atypical courses either above [6] or through [7,8] the muscle. Herein, we describe an unusual cadaveric case illustrating an atypical origin of the IGN.

## Case presentation

**Figure 1:**
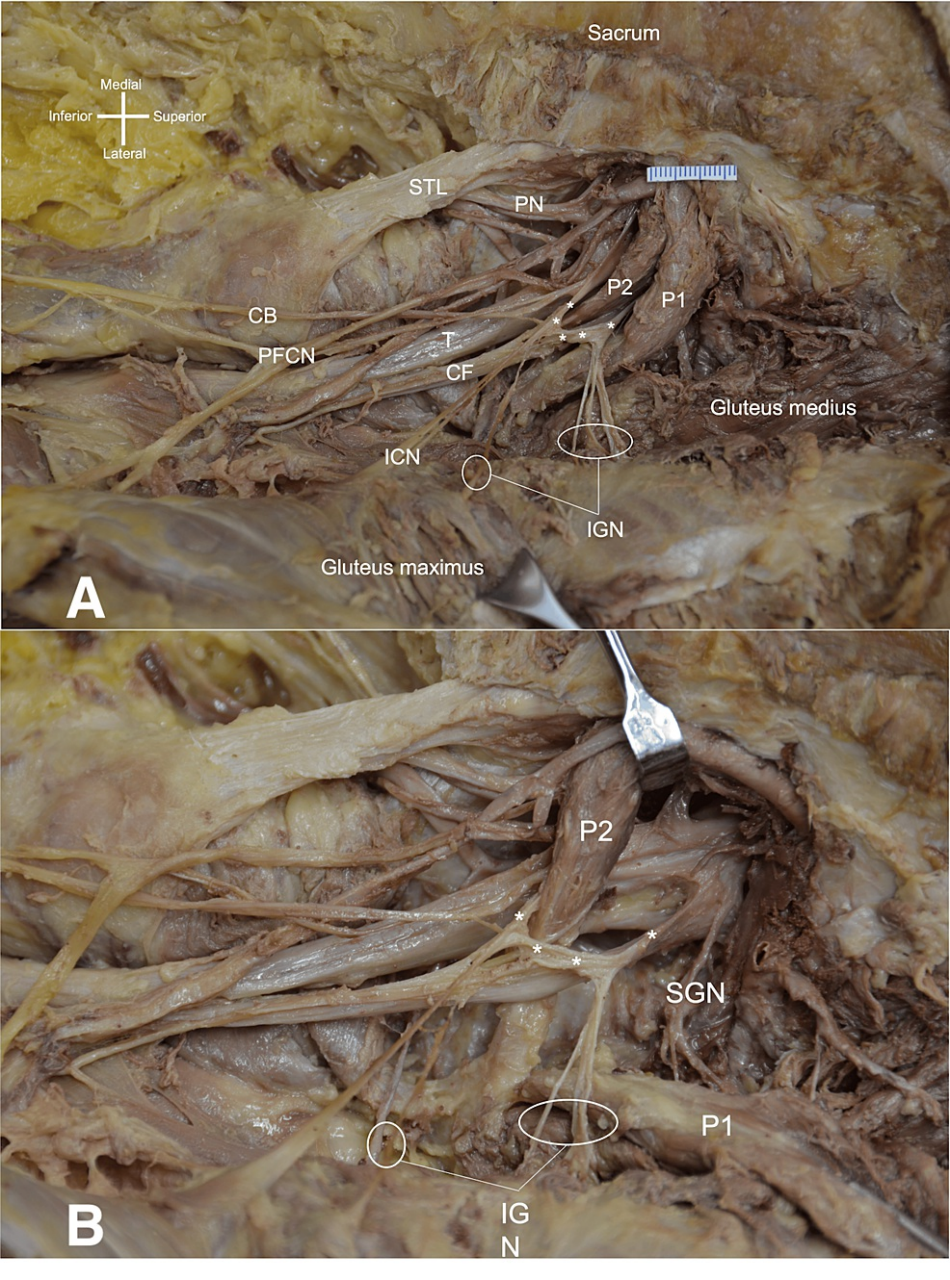
1A: Lateral retraction of GMax 1B: lateral retraction of PM Figure 1A: Right-sided gluteal dissection follows the retraction of the gluteus maximus laterally (under retractor). Note the splitting of the piriformis muscle into superior and inferior parts P1 and P2, respectively. Observe the inferior gluteal nerve branches (IGN) arising from the neural loop (*). The common fibular (CF) and tibial (T) components of the sciatic nerve, inferior clunial nerve (ICN), pudendal nerve (PN), sacrotuberous ligament (STL), cutaneous branch (CB), and posterior femoral cutaneous nerve (PFCN). Figure 1B: After lateral retraction of the P1 part of the piriformis muscle, noting the superior gluteal nerve (SGN), neural loop (*), P2 part of the piriformis muscle retracted inferiorly, and again, the inferior gluteal nerve branches (IGN).

During routine dissection of the right gluteal region of a male cadaver, 82 years old at death, an unusual formation of the IGN was observed. In the prone position, the skin and superficial fascia were removed with a scalpel. After dissecting the GMax from the sacrum, ilium, and sacrotuberous ligament, the muscle was reflected laterally, leaving only its attachment to the iliotibial tract and greater trochanter intact. The underlying gluteus medius (GMed) and PM were appreciated. At this point in the dissection, it was noted that the specimen had a split PM. The components of the SN (common fibular and tibial components) traveled through and inferior to the PM, respectively. Distal to the PM and approaching the deep surface of the GMax, a loop of nerve was formed by the components of the SN external to the PM (Figure 1a). From this nerve loop, four branches were given off that traveled to the deep surface of the GMax and innervated it. Each branch was, on average, 0.5 mm in diameter (range 2.5-0.7 mm). None of these muscular branches were found to pierce the muscle and enter the subcutaneous tissues. A fifth and more inferior branch traveled below the lower border of the GMax to ascend to the skin over the lower surface of the GMax as a cutaneous branch (i.e., inferior clunial nerve). A sixth branch left the nerve loop to join the posterior femoral cutaneous nerve (PFCN), and a seventh branch traveled medially to the skin, overlying the ischial tuberosity. The split components of the SN reunited just distal to the ischial tuberosity and traveled in a normal fashion to the posterior thigh and then popliteal fossa where they split into a lateral common fibular branch and medial tibial branch. The PFCN continued distally in a typical fashion to the skin over the posterior thigh and proximal popliteal fossa. Next, the superior head of the split PM was cut at its emergence from the greater sciatic foramen and then reflected laterally (Figure 1b). The superior gluteal nerve (SGN) was then seen to travel above the superior head of the PM to innervate the gluteus medius, minimus, and tensor fasciae latae muscles. No additional anatomical variations were identified in the gluteal or posterior thigh regions. Dissection of the contralateral side of this cadaver did not reveal a similar finding concerning the PM or nerves innervating the GMax.

The authors state that every effort was made to follow all local and international ethical guidelines and laws that pertain to the use of human cadaveric donors in anatomical research [9].

## Discussion

A variation of the relationship between nerves and muscles in the gluteal region is described herein, and appreciation of this variation is critical for doctors and allied health professionals to bear in mind when caring for a patient. Beaton and Anson proposed a six-category classification of the anatomical relationships between the SN and PM: (A) an undivided SN traveling below, (B) a split SN traveling through and below, (C) a split SN traveling above and below, (D) an undivided SN traveling through, (E) a split SN traveling above and through, and (F) an undivided SN traveling above the PM [10]. A review and meta-analysis showed that most individuals have Type A (prevalence approximately 83%) and that the prevalence of any form of SN-splitting by the PM (Types B, C, and E) is 15% according to the literature [3,11].

Studies describing the relationship of the IGN to the PM, however, suggest that atypical variants are both less common and under-reported. One review showed that the IGN traverses superior to the PM in 0.1-4.4% of individuals [6]. The present case, like others, describes an instance where the nerve pierces the PM directly [7,8]; another case describes its complete absence [5]. Notably, these variants of the IGN appear to be strongly associated with ipsilateral splitting of the SN by the PM, possibly because these abnormalities have a shared embryological origin [5,7,8].

Sumalatha et al. (2022) reported a case of an SN with a high division (i.e. the CFN and TN descended parallel to and independently of each other), and the IGN being absent [5]. They found that the CFN provided motor innervation to the GMax muscle in place of the missing IGN.

If the CFN passes inferiorly to PM (normal presentation), the IGN could arise from beneath it since the two nerves have common roots. Therefore, if there is an anatomical variation in which the CFN traverses over the PM superiorly, and depending on the proximity of the sacral and lumbar roots to that nerve, the IGN could also pass superior to the PM or even pierce it. Collins et al. (2022) theorized that physical characteristics, such as sex and height, could contribute to the variations in the height at which the SN splits into its components and the relative positioning of the SN with respect to the PM [12]. Future studies could investigate the extent to which variations in the CFN correlate with variations in the IGN.

As the neurovasculature weaves between the rotator muscles of the gluteal region, the nerves are susceptible to compression by those muscles. If the IGN is injured, clinically we would expect to see the weakness of lateral rotation, as this nerve innervates the GMax, a powerful lateral rotator. Some lateral rotation is preserved because the auxiliary external rotator muscles (i.e. PM, gemelli, obturator internus, quadratus femoris) would still be functional. Loss of sensation on the skin in the proximal femoral region is expected, as well [2].

There are few reports regarding the trajectory and variations of the PFCN, but there are some salient findings. Tunali, Cankara, and Albay (2016) reported that the PFCN could pass below the PM and have a communicating branch with the SN [13]. The existing literature agrees that when a split SN is present, the PFCN travels with the IGN to pierce the PM with CFN [14]. Kachniarz (2021) reported a high splitting pattern of the PFCN, giving off lateral and medial branches immediately distal to the PM that descend to innervate the thigh [15]. If it pierces the PM, the PFCN will exhibit the characteristic nerve entrapment seen when the IGN pierces the PM. However, if the PFCN splits proximal to the PM, the lateral or medial branches could be spared and relevant deficiencies would be expected [13,16].

## Conclusions

We have described an uncommon variant of the pelvic anatomy in a cadaveric specimen in which the piriformis split the sciatic nerve, and the IGN pierced that muscle. Atypical anatomy in this region can predispose individuals to pathological conditions, including nerve entrapment, and can increase the risk for iatrogenic injury during surgery and other procedures.
